# The Serine Protease CD26/DPP4 in Non-Transformed and Malignant T Cells

**DOI:** 10.3390/cancers13235947

**Published:** 2021-11-26

**Authors:** Guranda Chitadze, Ulrike Wehkamp, Ottmar Janssen, Monika Brüggemann, Marcus Lettau

**Affiliations:** 1 Unit for Hematological Diagnostics, Department of Internal Medicine II, University Hospital Schleswig-Holstein, Langer Segen 8-10, D-24105 Kiel, Germany; Guranda.Chitadze@uksh.de (G.C.); m.brueggemann@med2.uni-kiel.de (M.B.); 2 Department of Dermatology, University Hospital Schleswig-Holstein, Arnold-Heller-Str. 3, Bldg. C, D-24105 Kiel, Germany; uwehkamp@dermatology.uni-kiel.de; 3 Institute of Immunology, University Hospital Schleswig-Holstein, Christian-Albrechts University Kiel, Arnold-Heller-Str. 3, Bldg. U30, D-24105 Kiel, Germany; Ottmar.Janssen@uksh.de

**Keywords:** CD26, DPP4, dipeptidyl peptidase 4, T cells, cytotoxic granules, cutaneous T-cell lymphoma, mycosis fungoides, Sézary syndrome

## Abstract

**Simple Summary:**

The transmembrane serine protease CD26/Dipeptidylpeptidase 4 modulates T-cell activation, proliferation, and effector function. Due to their remarkable tumoricidal properties CD26-positive T cells are considered promising candidates for T cell-based immunotherapies while in cutaneous T cell lymphoma CD26/DPP4 expression patterns are established markers for diagnosis and possibly prognosis. With a focus on T cells, we review current knowledge on the regulation of CD26/DPP4 expression and release, its implication in T-cell effector function and the suitability CD26/DPP4 as a diagnostic and/or prognostic factor in T-cell malignancies.

**Abstract:**

CD26/Dipeptidylpeptidase 4 is a transmembrane serine protease that cleaves off N-terminal dipeptides. CD26/DPP4 is expressed on several immune cell types including T and NK cells, dendritic cells, and activated B cells. A catalytically active soluble form of CD26/DPP4 can be released from the plasma membrane. Given its wide array of substrates and interaction partners CD26/DPP4 has been implicated in numerous biological processes and effects can be dependent or independent of its enzymatic activity and are exerted by the transmembrane protein and/or the soluble form. CD26/DPP4 has been implicated in the modulation of T-cell activation and proliferation and CD26/DPP4-positive T cells are characterized by remarkable anti-tumor properties rendering them interesting candidates for T cell-based immunotherapies. Moreover, especially in cutaneous T-cell lymphoma CD26/DPP4 expression patterns emerged as an established marker for diagnosis and treatment monitoring. Surprisingly, besides a profound knowledge on substrates, interaction partners, and associated signal transduction pathways, the precise role of CD26/DPP4 for T cell-based immune responses is only partially understood.

## 1. Basic Features of CD26/DPP4

**Figure 1:**
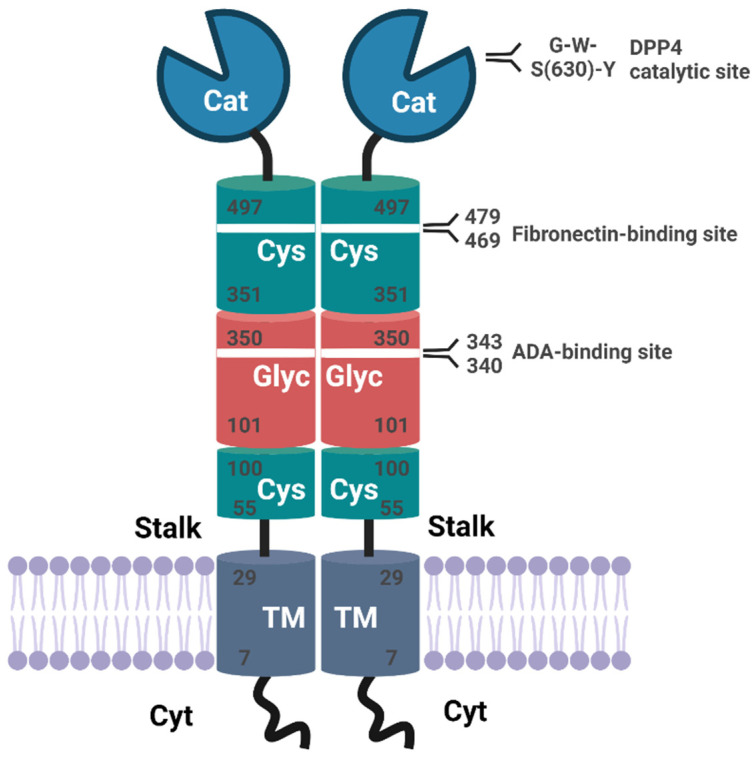
Schematic representation of DPP4 modular structure. Human CD26/DPP4 is a type 2 transmembrane serine protease comprising 766 amino acids (aa). The extracellular catalytic region (Cat, aa 506–766) with the catalytic triad H_740_, N_708_, and S_630_ is located at the C-terminus followed by a cysteine-rich region (Cys, aa 351–497), a glycosylated region (Glyc, aa 101–350), another short cysteine-rich region (Cys, aa 55–100), and a flexible stalk region (aa 30-48). The transmembrane domain (TM) encompasses aa 17–29 and only 6 amino acids constitute the N-terminal cytosolic (Cyt) part of the molecule. The extracellular part contains binding sites for interaction partners like adenosine deaminase (aa 340–343) or fibronectin (aa 469–479). Full-length CD26/DPP4 is suggested to be cleaved within its flexible stalk region to release catalytically active soluble CD26/DPP4 (figure adapted from [1]).

CD26/Dipeptidylpeptidase 4 (DPP4) is a 110 kDa type 2 transmembrane glycoprotein that belongs to the S9 protease family of prolyl oligopeptidases (EC 3.4.14.5) that cleaves off N-terminal dipeptides with penultimate prolines or alanines. The extracellular part of the protein comprises a C-terminal catalytic domain with the catalytic triad S_630_, N_708_, and H_740_, a cysteine-rich area and a glycosylated region that is linked to the transmembrane region via a flexible stalk. The extracellular domain also contains binding sites for example for adenosine deaminase (ADA) and components of the extracellular matrix like fibronectin. Of note, only six N-terminal amino acids are predicted to extend into the cytosol. The active site S_630_ is flanked by the classic serine peptidase motif G-W-S_630_-Y-G-G-Y-V (Figure 1) (reviewed in [1,2,3]).

Moreover, the C-terminal region, an extracellular propeller loop and the transmembrane region might contribute to CD26 dimerization and enzymatic activity [4]. In addition, dimerization might also modulate protein interactions of the cytoplasmic tail. For example, only dimeric CD26 interacts with CARMA-1 [5]. CD26/DPP4 is rather ubiquitously expressed on blood cells, fibroblasts, mesothelial, epithelial, and endothelial cells and is detected in several organs including placenta, kidney, intestine, prostate, gall bladder, pancreas, and liver [6,7,8]. In the immune system, CD26/DPP4 is expressed on dendritic cells, activated B cells, natural killer (NK) cells, T cells, and CD34^+^ progenitor cells (reviewed in [1]).

## 2. Soluble CD26/DPP4

A catalytically active soluble form of CD26/DPP4 (sCD26/DPP4) that lacks the intracellular and transmembrane domain is released from the plasma membrane [9] and can be detected in serum, saliva, cerebrospinal and seminal fluid, and bile [10]. The enzymatic CD26/DPP4 activity in serum has been mainly attributed to soluble sCD26 [9] and serum levels of DPP4 activity and/or sCD26 protein were associated with diverse diseases including autoimmunity, infections, autoimmunity, and malignancies (reviewed in [10,11]). In vivo analyses in mice showed that serum sCD26/DPP4 originates from endothelial and bone marrow-derived cells [12]. It is generally accepted that sCD26/DPP4 originates from the proteolytic cleavage of full length CD26/DPP4 and numerous proteases mediating the release of sCD26/DPP4 have been suggested. The matrix metalloproteases 1 (MMP1), MMP2, and MMP14 have been functionally associated with the shedding of sCD26/DPP4 from smooth muscle cells, while MMP9 has been implicated in the release of the soluble form from adipocytes [13] and MMP10/13 from ovarian cancer cells [14]. Apart from metalloproteases, the serine protease kallikrein 5 (KLK5) was suggested to facilitate sCD26/DPP4 release from TH_17_ cells [15].

Taken together, there are likely differentially regulated, cell-type specific mechanisms of (s)CD26/DPP4 release that are even more complex when considering potential interrelationships with other protease systems.

## 3. Intracellular CD26/DPP4

**Figure 2:**
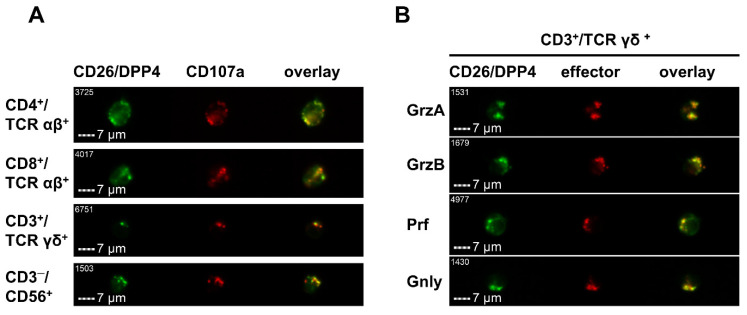
Intracellular storage of CD26 in cytotoxic effector lymphocytes. Co-localization of intracellular CD26 with (**A**) the lysosomal and degranulation marker protein CD107a (Lamp-1) in CD4^+^/TCR αβ^+^, CD8^+^/TCR αβ^+^, CD3^+^/TCR γδ^+^, and CD3^−^/CD56^+^ cells and (**B**) the cytotoxic effector proteins granzyme A (GrzA), granzyme B (GrzB), perforin (Prf), and granulysin (Gnly) in CD3^+^/TCR γδ^+^ cells.

Of note, CD26/DPP4 is mainly located on the cell surface but based on the observation that both CD26^+^ and CD26^−^ T cells exhibit comparable levels of CD26/DPP4 mRNA and overall protein, an intracellular pool of CD26/DPP4 in T cells was suggested [16]. Furthermore, CD26/DPP4 was also detected in lysosomal organelles of hepatocytes, endothelial cells, and Kupffer cells in rat liver sections [17] and in secretory granules of A-cells in pig pancreatic islets by immunoelectron microscopy [18]. Moreover, CD26/DPP4 was identified in proteomic screens of enriched organelles from the leukemia/lymphoma NK cell line YTS [19] and of lysosomal effector compartments from non-transformed human T cells [20,21]. Additional analyses confirmed the intracellular storage of CD26/DPP4 in secretory granules of human cytotoxic lymphocytes including CD8^+^ α/β T cells, γ/δ T cells, and NK cells, and also in a small fraction of cytotoxic CD4^+^ α/β T cells, where it co-localizes with effector proteins like granzymes, perforin, and granulysin. Figure 2A exemplarily shows the colocalization of intracellular CD26/DPP4 with the lysosomal and degranulation marker protein CD107a in CD4^+^/TCR α/β^+^, CD8^+^/TCR α/β^+^, CD3^+^/TCR γ/δ^+^, and CD3^−^/CD56^+^ cells and Figure 2B with the cytotoxic effector proteins granzyme A, granzyme B, perforin, and granulysin in CD3^+^/TCR γ/δ^+^ cells.

In response to appropriate stimuli, intracellular CD26/DPP4 is rapidly translocated to the cell surface and this activation-induced degranulation is accompanied by the release of proteolytically active sCD26/DPP4. Effector lymphocytes are thus clearly an additional source of sCD26/DPP4 [22,23]. Moreover, these results might provide additional insight into disease-associated alterations of sCD26/DPP4 serum levels and indicate a so far unknown role of CD26/DPP4 in T cell-mediated cytotoxicity. In addition, full length CD26/DPP4 might be released in association with extracellular vesicles (EV). Here, CD26/DPP4 was detected on plasma exosomes of head and neck squamous carcinoma patients that were derived from both tumor cells and T cells [24]. Of note, in this scenario full-length CD26/DPP4 might ligate potential interaction partners in an autocrine and also paracrine manner. In addition, EV-released CD26/DPP4 is considered proteolytically active [25]. As an example, exosomes isolated from the plasma of acute myeloid leukemia (AML) patients inhibited colony formation of normal hematopoietic progenitor cells and were thus implicated in the suppression of hematopoiesis and to contribute to AML-associated cytopenia. These exosomes were characterized by the expression of enzymatically active CD26/DPP4 and DPP4 inhibition abrogated the effects on HPC colony formation [25]. However, in most contexts the functional role of EV-associated CD26/DPP4 is poorly characterized.

The sorting mechanisms that target CD26 to intracellular storage granules and to exosomes have not been analyzed so far. However, T-cell granules have been referred to as secretory lysosomes, bifunctional organelles that serve both as a degradative and a storage compartment that allows for regulated mobilization and fusion with the plasma membrane in response to appropriate stimuli. Transmembrane components are thus locally exposed on the cell surface while soluble effector molecules are secreted into the immunological synapse that is formed between target and effector cell [26,27]. As multivesicular bodies, these effector compartments contain intraluminal vesicles (ILV) that are released upon fusion with the plasma membrane and are then referred to as exosomes [28]. Since T-cell degranulation leads to an increase of CD26 surface expression [22], CD26 is likely located in the outer membrane of the effector granules and since it is also released as a transmembrane component of exosomes it must be additionally sorted to ILVs. The subcellular site of the proteolytic conversion of transmembrane CD26 to its soluble form is not known. Shedding of CD26 might either take place on the cell surface or within intracellular storage granules and precede degranulation to readily release the soluble form upon activation. The sorting mechanisms that target CD26 first to intracellular storage granules and subsequently to exosomes have not been characterized so far. Protein sorting to the lumen of MVB, the outer membrane, or subsequently to ILV relies on multiple different mechanisms. For example, granzymes and lysosomal hydrolases are targeted to effector granules via the mannose-6-phosphate (M6P) pathway [29]. Here, a M6P tag is added to N-linked oligosaccharides in the cis-Golgi network (CGN). This tag is then recognized by transmembrane M6P receptors in the trans-Golgi network that bind to cargo proteins on the luminal side of the membrane and to adaptins in forming clathrin coats on the cytosolic side to allow for packaging into transport vesicles for the delivery to lysosomes (reviewed in [30]). Interestingly, in activated T cells CD26 is phosphorylated at mannose residues in its extracellular part and interacts with the M6P receptor although this was functionally associated with endocytosis rather than direct lysosomal trafficking from the trans-Golgi network (TGN) [31].

Like supposed for CD26, the transmembrane death factor FasL is also associated with the outer membrane of secretory lysosomes to end up on the cell surface after fusion with the plasma membrane as well as with the membrane of intraluminal vesicles to be released in association with exosomes [32,33]. The sorting of FasL is quite complex and involves SH3 domain-mediated binding of tyrosine kinases and proteins of the pombe/cdc15 homology family to an intracellular proline-rich domain and tyrosine phosphorylation at phosphorylation sites within the cytoplasmic tail [32,34,35,36,37]. Further sorting to intraluminal vesicles requires mono-ubiquitination at an intracellular KKR motif and the ESCRT machinery [32]. However, the sorting of other exosomal proteins does not rely on ESCRT proteins and involves different post-translational modifications (PTM) like SUMOylation, acetylation, or glycosylation (reviewed in [38]). Of note, the cytoplasmic tail of CD26 comprises only six amino acids (MKTPWK) and characteristic PTM have not been described so far.

## 4. Functions of CD26/DPP4

### 4.1. General Functions

Considered a multifunctional “moonlighting” protein, the serine protease CD26/DPP4 has been implicated in numerous biological processes [39]. CD26/DPP4 effects are either dependent or independent of its enzymatic activity and are exerted by the transmembrane protein and/or the soluble form. CD26/DPP4 cleaves off N-terminal dipeptides with penultimate prolines or alanines and CD26/DPP4 substrates include gastrointestinal hormones, growth factors, chemokines, neuropeptides, and regulatory peptides including neuropeptide Y (NYP), glucose-dependent insulinotropic polypeptide (GIP), glucagon-like peptide 1/2 (GLP-1/2), stromal cell-derived factor-1α (SDF-1α, CXCL12), CCL11/eotaxin-1, erythropoietin, granulocyte macrophage colony-stimulating factor (GM-CSF), CXCL10, and CCL5/RANTES (regulated upon activation normal T cell expressed and secreted). Here, the functional effects of CD26/DPP4-mediated cleavage include the inactivation of substrates, the modulation of receptor specificities or the generation of bioactive peptides (reviewed in [2]).

### 4.2. Interaction Partners and Implications for T-Cell Function

Interaction partners of CD26/DPP4 include for example adenosine deaminase [40], caveolin-1 [41], CD45 [42,43], CXCR4 [44], collagen (especially 1 and 3, [45]), fibronectin [46], glypican 3 [47], caspase-recruitment domain containing protein 1 (CARMA1, [48]) and M6P/IgFr2 ([31,49]) (for additional interaction partners refer to [1]). Moreover, CD26/DPP4 was also suggested as a (co-)receptor for SARS-CoV-2, the virus causing the respiratory syndrome COVID-19 [50] since the closely related virus MERS-CoV utilizes CD26/DPP4 for viral entry [51].

As a T cell surface molecule, CD26/DPP4 has been functionally associated with the modulation of T-cell activation and proliferation [52]. Early reports demonstrated that antibody-mediated ligation of CD26/DPP4 induced tyrosine phosphorylation of signaling molecules such as c-Cbl, MAP kinase, ERK1/2, Zap70, Lck, and CD3ζ [53]. However, despite recent advances the molecular details of the impact of CD26/DPP4 on T-cell activation and associated functional consequences for T cell-mediated immune responses are still far from being understood.

#### 4.2.1. Adenosine Deaminase

**Figure 3:**
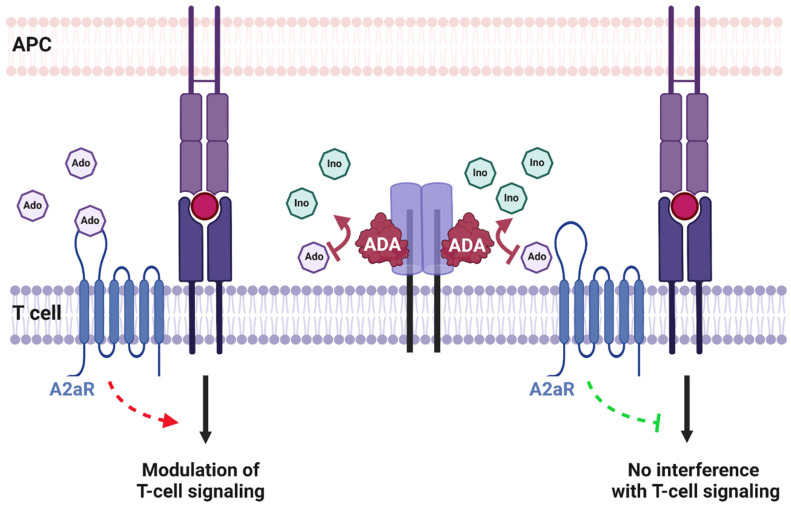
Interaction of CD26 with adenosine deaminase (ADA). Extracellular adenosine (Ado) interferes with T-cell receptor signaling by binding to the adenosine receptor 2a (A2aR) expressed on T cells (A). ADA anchored to CD26 metabolizes immunosuppressive adenosine to inosine (Ino) and in this way facilitates T-cell activation and differentiation to effector/memory T cells.

As mentioned, CD26/DPP4 was shown to interact with ADA [40]. ADA is a 41 kDa protein that catalyzes the conversion of adenosine and 2′-desoxyadenosine to inosine and 2′-desoxyinosine by deamination. ADA deficiency causes early-onset severe combined immunodeficiency (SCID), which is characterized by the loss of functional T, B, and NK cells, impaired cellular and humoral immunity, and susceptibility to infections [54]. Extracellular adenosine interferes with TCR signaling by binding to the adenosine receptor 2a (A2aR) expressed on effector T cells [55]. CD26/DPP4 was identified as an ADA-anchoring protein and in this way CD26/DPP4 accumulates ADA at the cell surface. ADA then metabolizes immunosuppressive adenosine and thus positively modulates T-cell activation (Figure 3) [56].

Depending on its binding to cell surface CD26/DPP4, ADA enhances the differentiation of naïve T cells to effector, memory, and regulatory T cells [57]. Moreover, within the immunological synapse formed between dendritic cells and T cells, ADA interactions with A1R and A2bR (DC side) and CD26/DPP4 (T-cell side) mediate costimulatory signals and promote T-cell proliferation and differentiation [58]. ADA-associated co-stimulation also facilitates an increase in T helper type (Th) 1 cells and pro-inflammatory cytokines including interferon-γ (IFN-γ), interleukin-6 (IL-6), and tumor necrosis factor-α (TNF-α) [58]. Moreover, ADA treatment of CD26^+^/TCR Vδ2^+^ T cells increased cytokine responsiveness and IFN-γ production [59]. However, studies employing mutant CD26/DPP4 that is unable to bind ADA or that is catalytically inactive indicate that CD26/DPP4 modulates T-cell proliferation independent of ADA binding and substrate cleavage [60].

#### 4.2.2. CD45

CD26/DPP4 also interacts with the cytoplasmic region of the tyrosine phosphatase CD45 to enhance T-cell receptor signaling. Antibody-induced ligation of CD26/DPP4 on T cells resulted in an internalization of CD26/DPP4, a decrease in CD45RO surface expression, increased Lck kinase activity, and enhanced phosphorylation of the CD3ζ chains [42,43]. Subsequent studies revealed that ligation of CD26/DPP4 triggers the recruitment of CD26/DPP4 molecules to rafts where it colocalizes with CD45 as a prerequisite for CD26/DPP4-modulated tyrosine phosphorylation [42].

#### 4.2.3. Caveolin-1

**Figure 4:**
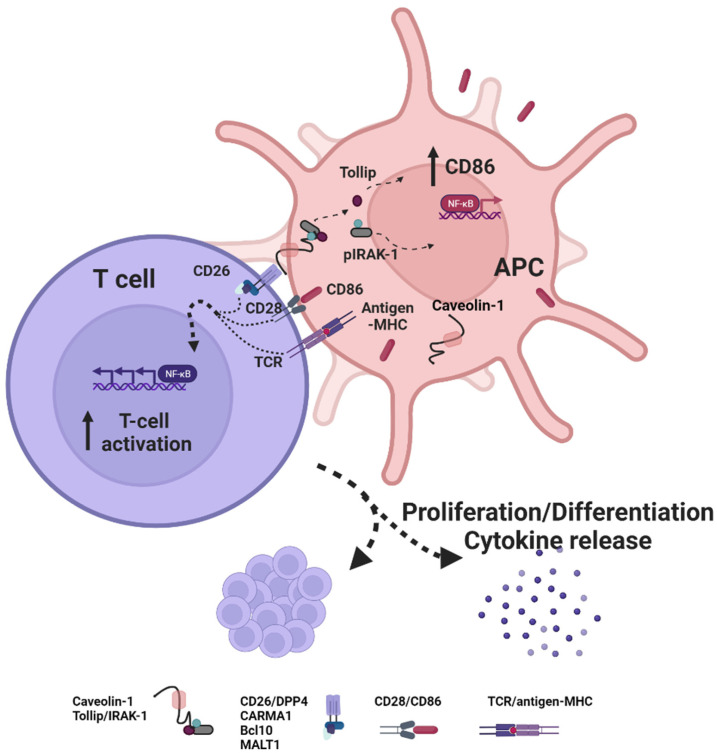
Interaction of CD26 with caveolin-1. Caveolin-1 on the surface of antigen-presenting cells (APC) binds to CD26 on the T-cell surface. This interaction induces an increase of the expression of the costimulatory ligand CD86 on APC thus enhancing T-cell co-stimulation by binding to CD28. In addition, caveolin-1-mediated CD26 ligation results in the recruitment of a complex composed of CD26, CARMA1, Bcl10, and MALT1 to lipid rafts and subsequent NF-κB activation in a TCR/CD3-dependent manner. CD26-mediated co-stimulation enhances the T-cell proliferation and effector function with respect to cytotoxic properties and cytokine release.

T-cell CD26/DPP4 interacts with caveolin-1 on antigen-presenting cells and induces an increase in CD86 expression to facilitate T-cell co-stimulation [41]. In addition, CD26/DPP4 ligation by caveolin-1 enhances T-cell proliferation and NF-κB activation in a TCR/CD3-dependent manner and is associated with the recruitment of a complex consisting of CD26/DPP4, CARMA1, Bcl10, and IκB kinase to lipid rafts (Figure 4) [5].

Interfering with the Caveolin-1/CD26 interaction with a soluble caveolin-1-Ig fusion protein induces anergy in CD4^+^ T cells [48]. In addition, CD26/DPP4-mediated co-stimulation of anti-CD3-activated CD8^+^ T cells enhances the cytotoxic properties compared to CD28-stimulated T cells and leads to increased expression of TNF-α, IFN-γ, granzyme B (GrzB), and soluble Fas ligand (FasL) [61].

### 4.3. Effect of DPP4 Inhibitors on T-Cell Function

Interestingly, DPP4 inhibitors including Sitagliptin, Teneligliptin, and Anagliptin that are used for the treatment of type 2 diabetes mellitus (T2DM, see below) were shown to have a negative impact on T-cell receptor signaling, T-cell activation, and proliferation in ex vivo analyses [62]. Moreover, in new-onset diabetic mice as an in vivo model system for autoimmunity DPP4 inhibition increased the proportion of T regulatory cells and even reversed the disease [63]. Altogether, this argues for an effect of CD26/DPP4 enzymatic activity on T-cell function. Of note, mice with genetic elimination of CD26/DPP4 or treated with DPP4 inhibitors nevertheless displayed robust primary and secondary antibody responses to T-dependent antigens, indicating that CD26/DPP4 might not be essentially required for mounting a T-cell directed immune response despite its proven role in T-cell activation [64]. However, available DPP4 inhibitors differ in their specificity for DPP4 and some also inhibit the closely related DPP8 and DPP9. In in vitro models of T-cell activation, highly selective DPP8/9 inhibitors attenuated T-cell proliferation and IL2-release while the specific inhibition of DPP4 did not affect T-cell activation indicating that previously observed immunological effects of several DPP4 inhibitor compounds may be due to off-target inhibition of DPP8/9 [65].

### 4.4. CD26-Mediated Substrate Cleavage

Thus, the exact role of CD26/DPP4 in T-cell dependent immune responses and the most probably differential impact of different CD26/DPP4-associated signaling pathways might be rather context-dependent and far more complex than initially anticipated. As mentioned, CD26/DPP4 substrates include a wide array of cytokines and chemokines that are either activated, inactivated, or modulated with respect to receptor specificity by CD26/DPP4-mediated cleavage [2]. Thus, the effect of CD26/DPP4 on chemokine/cytokine networks that potentially regulate T-cell function is remarkably complex.

#### 4.4.1. GIP/GLP-1

As an example, CD26/DPP4 inactivates the insulinotropic incretin hormones GIP and GLP-1 by proteolysis. GIP and GLP-1 are released upon glucose intake and facilitate insulin secretion. The half-life of incretin hormones, however, is limited to only a few minutes due to CD26/DPP4-mediated cleavage. CD26/DPP4 inhibition prolongs the incretin effect and improves glucose tolerance. Commercially available CD26/DPP4 inhibitors are thus widely employed as an accompanying therapy in the treatment of diabetes mellitus type 2 [66]. Of note, blood glucose levels also affect T-cell activation, differentiation, and memory formation and glucose availability is locally limited at sites of infection/inflammation or in the tumor microenvironment (reviewed in [67,68]). For instance, in virus-specific T cells, reduced glucose levels in vitro significantly reduced cytokine production and cytotoxic effector potential but increased expression of CD69 and CD103 that are functionally linked to tissue retention [23,69]. Recent in vivo data suggested that sCD26/DPP4 secreted by bone marrow-derived cells might not contribute to systemic glucose control and affects plasma levels of GIP only and not of GLP-1/2 [12]. However, the effect of CD26/DPP4-deficiency or inhibition in hematopoietic cells on the regulation of blood glucose levels during an immune response to infections as well as in the context of autoimmunity or T-cell activating or chimeric antigen receptor (CAR) T-cell-based immunotherapies of cancer malignancies has not been addressed yet.

#### 4.4.2. Chemokines

CD26/DPP4 also cleaves several chemokines including for example CXCL12 (SDF-1α), CXCL10, CCL5/RANTES, and CCL11/eotaxin-1 (additional substrates reviewed in [2]). CXCL12 is a chemoattractant for lymphocytes, monocytes, and CD34^+^ hematopoietic precursor cells that express receptor CXCR4 [70]. CD26/DPP4-mediated removal of the two N-terminal amino acids converts full-length CXCL12 (1–67) to truncated CXCL12 (3–67) and completely revokes its chemotactic properties [71]. CXCL12 (3–67) retains significant binding affinity for the receptor and thus antagonizes the binding of biologically active CXCL12 (1–67) [72]. Likewise, CD26/DPP4-mediated processing of CXCL10 results in the generation of an antagonist form of the chemokine that inhibits T-cell and NK cell migration. The in vivo inhibition of CD26/DPP4 in humans can thus preserve the bioactive form of CXCL10. Here, DPP4 inhibitors might be employed as therapeutic immune modulators that affect T- and NK cell trafficking [73]. Likewise, CD26/DPP4 processes CCL5 that is released by activated T cells. In its full-length form, CCL5 (1–68) recruits innate immune cells to the site of inflammation whereas its chemotactic properties are drastically reduced upon CD26/DPP4-mediated conversion to CCL5 (3–68) [74]. Of note, the CD26-mediated cleavage of CCL5 might also enhance T-cell migration in certain scenarios [75]. For additional information on the role of CD26-mediated processing of chemokines in malignancies we recommend the recent in-depth review by De Zutter and colleagues [76].

#### 4.4.3. HMGB1

High mobility group box 1 protein (HMGB1) is a ubiquitously expressed cytokine that is released upon injury and infection and functions as an alarmin to evoke inflammatory and regenerative responses. It modulates tissue regeneration, angiogenesis [77], and induces pro-inflammatory effects for example upon release from macrophages [78,79]. Furthermore, the release of HMGB1 from NK cells was shown to facilitate dendritic cell activation [80] and chemotaxis [81]. In breast cancer patients, HMGB1 released from dying tumor cells facilitates the anti-tumor immune response by ligation of Toll-like receptors 2 and 4 on dendritic cells to enhance processing and presentation of tumor antigens [82]. In addition, NK cell-derived HMGB1 also induces a distinct form of necrotic cell death in cancer cells that targets mitochondrial energy metabolism by blocking pyruvate kinase isoform M2 thus inhibiting glucose-dependent aerobic respiration ultimately killing cancer cells by restricting their energy supply to glycolysis [83,84]. Interestingly, HMGB1 was also shown to have intrinsic antimicrobial activity [85]. Given its alarmin functions as well as its immunomodulatory and direct cytotoxic effector properties, HMGB1 subcellular location, release, and activity is regulated on several levels [86]. Interestingly, CD26/DPP4 cleaves HMGB1 at its N-terminal region and modulates HMGB1 function. As an example, CD26/DPP4-mediated HMGB1 processing inhibited HMGB1-induced endothelial cell migration. Moreover, this truncated form of HMGB1 was also detected in the serum of T2DM patients and the application of DPP4 inhibitors enhanced levels of full-length HMGB1 [87]. However, the effects of CD26/DPP4-mediated HMGB1 processing on its anti-bacterial and anti-tumor effects and its regulated release have not been analyzed so far.

Taken together, the impact of CD26/DPP4-mediated substrate processing on immune cells is quite complex with diverse functional outcomes. However, especially the role of lymphocyte-derived sCD26/DPP4 at the sites of immune cell activation and effector function on the processing of its substrates and associated functional consequences yet remain to be addressed.

### 4.5. CD26 as a T-Cell Marker Protein

Despite these open questions with respect to involved signaling pathways, CD26/DPP4 expression was clearly correlated with T-cell function, thus making it an interesting marker protein. It has been noted that increased CD26/DPP4 expression on CD4^+^ T cells correlates with a TH_1_-like phenotype whereas lower CD26/DPP4 expression was mainly seen on TH_2_-like CD4^+^ T cells [88]. After anti-CD3 antibody-mediated in vitro expansion, CD26^+^ T cells were shown to express higher levels of IL-2, IFN-γ, IL-6, IL-17, IL-22, IL-23 receptor (IL-23R), CCR6 and CD161 compared to CD26^low^ cells [89]. Among human peripheral blood mononuclear cells (PBMCs), CD26^hi^/CD8^+^ T cells are mainly CD26^hi^/CD28^+^/CCR7^−^/CD45RA^−^ effector memory cells [61]. Furthermore, high CD26/DPP4 expression along with low CD94 expression defines transcriptionally distinct TCR Vδ2^+^ T cells. Exposure of CD26^hi^/CD94^low^ TCR Vδ2^+^ T cells to phosphoantigen in the presence of IL-23 and simultaneous ligation of CD26/DPP4 with an agonistic antibody enhances the cytotoxic potential rendering them ideal targets for immunotherapeutic expansion and adoptive transfer [59]. In another analysis, the characterization of CD26^−^ lymphocyte subsets and those with an intermediate (CD26^int^) and high (CD26^hi^) CD26/DPP4 expression revealed striking differences in anti-tumor properties. Here, CD26^hi^/CD4^+^ T cells display a potent effector function against solid tumors. CD26^hi^/CD4^+^ T cells expressed high levels of cytotoxic effector molecules including Perforin, GrzA, GrzB, and GrzK and displayed an elevated expression of the chemokines MIP-1β (macrophage inflammatory protein-1β) and RANTES and of the cytokines IFN-γ, TNF-α, IL-2, IL-17, and IL-22 with a high fraction of cells co-secreting 4–5 cytokines simultaneously [90,91]. Of note, despite the expression of IL-17A, CD26^hi^/CD4^+^ T cells seem to be epigenetically and transcriptionally distinct from TH_17_ cells [90]. In addition, CD26^hi^ T cells display a characteristic chemokine receptor profile (CXCR3, CCR6, CD161) and are characterized by profound cytotoxicity, resistance to apoptosis, and enhanced stemness [90,91]. In accordance with these findings, CD26^hi^/CD4^+^ T cells genetically engineered to express a CAR recognizing mesothelin, an antigen overexpressed in mesothelioma, displayed an outstanding capacity to traffic to, survive in, and regress solid tumors in NSG xenograft mesothelioma models. In this scenario, the anti-tumor response of CD26^hi^ T cells was superior to other CD4 T-cell subsets such as TH_17_ and bulk CD4^+^ T cells and did not require CD8^+^ T cells indicative of a direct tumor-lysing capacity [90]. Interestingly, preliminary data also indicate that treatment with the CD26/DPP4 inhibitor sitagliptin impaired the migration of CD26^hi^ mesothelin-specific CAR T cells, thus limiting tumor infiltration by CD26^hi^ T cells resulting in impaired tumor control. Of note, in this scenario sitagliptin did not impair the overall ability of T cells to respond to antigen [92]. Given these results, the monitoring and characterization of tumor-infiltrating CD26^+^ T cells at the onset and during immunotherapy might help to assess its value as a predictive marker. In the context of immune checkpoint blockade where the presence of tumor-specific T cells is a prerequisite for an efficient response to therapy, patients who benefited from treatment displayed an increased proportion of CD26^hi^ tumor-infiltrating lymphocytes (TIL) prior to and following therapy [93]. Moreover, upon immune checkpoint blockade in mice, CD26/DPP4 is upregulated only in TIL from those animals that mounted an efficient anti-tumor response. However, more clinical studies are needed to address the role of CD26/DPP4 expression in TIL especially with respect to their role in the induction of effective immune responses and durable remissions following cancer immunotherapy and to unravel the underlying mechanisms in detail.

Of note, immunomodulatory effects also play a crucial role when considering the efficacy of T cell-based immunotherapies. Here, the blockade of the regulatory T cells (Tregs) is generally considered to be beneficial for the treatment outcome [94].

Recent analyses show that CD26 is present on FoxP3-expressing activated CD4^+^ effector T cells but absent from Tregs or expressed at reduced levels. Since this CD26^−/low^ phenotype is stable it allows for the differentiation of Tregs from effector T cells in analyses or enrichment protocols [95]. This reduced expression or absence in Tregs might also partially account for their functional properties. Since cell surface CD26 binds ADA, reduced CD26 levels might increase levels of immunosuppressive adenosine and adenosine in turn has been shown to increase the numbers of Tregs and to further promote their immunoregulatory activity [96]. As already mentioned, the interaction of CD26 with caveolin-1 increases the expression of costimulatory CD86 molecules in APCs, whereas Tregs downmodulate both CD80 and CD86 expression via a CTLA4-dependent mechanism [97]. Of note, CD86 is upregulated in response to proinflammatory stimuli and preferentially binds to activating CD28, whereas CD80 appears to be rather specific for the inhibitory receptor CTLA-4 [98,99]. Therefore, in this scenario, the low expression of CD26 on Tregs might contribute to low levels of proinflammatory CD86 on APCs which results in insufficient activation of antigen-primed naïve T-cells ultimately inducing anergy.

## 5. CD26/DPP4 in T-Cell Malignancies

Given its diverse functions, it is not surprising that dysregulation of the expression or the enzymatic activity of CD26/DPP4 might contribute to cancer development [100].

Although we focus on T-cell malignancies in the following, it should be mentioned that several solid tumors including for example colorectal cancer (CRC), hepatocellular cancer (HCC), malignant mesothelioma and renal cell carcinoma (RCC) express CD26/DPP4. For instance, high expression levels of CD26/DPP4 were significantly associated with advanced tumor stages in a cohort of CRC patients and with reduced overall and disease-free survival [101]. Moreover, in preclinical model systems of RCC and malignant mesothelioma, the presence of blocking anti-CD26/DPP4 antibodies inhibited tumor growth and metastasis. Meanwhile, the humanized anti-CD26/DPP4 mAb YS110 (NCT03177668) has already been tested in humans for its safety and efficacy for the treatment of aggressive malignant mesothelioma, a disease with otherwise still very limited treatment options [102,103]. CD26 has also been functionally associated with metastasis. The escape of cancer cells from the primary tumor, entry into the blood stream, and extravasation to form new tumor colonies in secondary organs require the action of a wide variety of cytokines, chemokines, ligands, receptors, and many of those are either substrates or binding partners of CD26 [104,105]. However, some cancer types are characterized by a low expression of CD26, and studies even indicated that induced expression of CD26 in those malignant cells reverted the malignant phenotype and improved control of metastasis, invasion, and proliferation [106,107]. The functional role of CD26 in malignant cells appears to depend on tumor localization, cell type, and the local microenvironment and CD26 can be regarded as both a tumor suppressor and as a marker of tumor aggressiveness.

Early studies also suggested a role of CD26/DPP4 in hematological malignancies. CD26/DPP4 is expressed on aggressive T-cell hematological malignancies such as T-cell lymphoblastic lymphoma (T-LBL) and T-cell acute lymphoblastic leukemia (T-ALL) and was associated with poor survival [108]. At the functional level, antibody- or siRNA-mediated inhibition of CD26/DPP4 in the CD26^+^ T-ALCL (anaplastic large cell lymphoma) line Karpas-299 resulted in decreased adhesion properties in vitro and improved survival in vivo [109].

As mentioned, pharmacologic inhibitors of CD26/DPP4 have been in clinical use for over a decade to improve glucose tolerance in diabetic patients [66]. While distinct reports indicate an increase in incidence rates of selected cancers [110,111,112], other studies argue against an association of tumor incidence and CD26/DPP4 inhibition and some analyses even indicate that CD26/DPP4 inhibition might instead improve outcomes related to certain tumor types [113,114,115,116,117,118]. However, current clinical evidence is not sufficient to evaluate potential tumor-promoting or tumor-suppressive effects of CD26/DPP4 inhibition and functional explanations are still missing. Here, especially effects of CD26/DPP4 inhibition on the immune system are discussed to potentially affect tumor incidence rather than direct pro-tumorigenic effects on healthy cells [1]. Apart from its potential role as a therapeutic target, levels of CD26/DPP4 expression and sCD26/DPP4 release are discussed as putative diagnostic and prognostic markers.

Although CD26/DPP4 surface expression in B-ALL, AML, and T-ALL was comparable to levels observed in patients with non-leukemic hematologic alterations or in healthy controls, plasma CD26/DPP4 activity was significantly higher in leukemia patients [119]. However, the diagnostic and prognostic value of (s)CD26/DPP4 in most hematologic malignancies is still a matter of debate. Nevertheless, numerous studies analyzed CD26/DPP4 cell surface expression and levels of sCD26/DPP4 in the serum of patients with cutaneous T-cell lymphoma (CTCL) including the most frequent CTCL mycosis fungoides (MF) and Sézary syndrome (SS) [120]. It has been noted that serum sCD26/DPP4 levels were significantly lower in patients with MF compared to healthy controls [121], and plasma CD26/DPP4 activity was decreased in patients with SS or MF compared to healthy individuals [120]. In addition, it has been reported that CD26/DPP4 expression predominates in the early stages of MF [122] and is lost with disease progression [121,122,123,124]. Likewise, frequencies of CD26^−^/CD4^+^ T cells are significantly increased in SS [122]. This loss of CD26/DPP4 expression is also observed in the CD8^+^ T-cell compartment in both MF [125] and SS [126]. Counts of these atypical CD26^−^ lymphoid cells referred to as Sézary cells are meanwhile considered a quite sensitive and specific parameter for the early detection of SS and of MF. Thus, CD26/DPP4 is meanwhile included in validated EuroFlow panels for the immunophenotyping of T-cell chronic lymphoproliferative diseases [127,128,129,130,131]. Although other markers can be overexpressed (e.g., PD-1) [132] or lost (e.g., CD7) [133] in CTCL, CD26/DPP4 is meanwhile one of the most established marker proteins for SS and MF markers [130,131]. The frequency of CD26^−^/CD4^+^ T cells displays the extent of blood involvement and can be used for monitoring disease status, treatment response, and to assess prognosis [134] and is thus recommended for staging by the EORTC/ISCL (European Organization for Research and Treatment of Cancer/International Society for Cutaneous Lymphoma) and as a diagnostic criterion for SS syndrome [135]. However, it has been proposed recently to evaluate the degree of blood involvement based on absolute counts of atypical lymphocytes [136]. Of note, counts of CD26^−^/CD4^+^ T cells are not sufficient for CTCL diagnosis since not all CTCLs display a loss of CD26/DPP4 (and CD7) expression. Moreover, loss of CD26/DPP4 expression might also be observed under physiological conditions or in non-malignant pathological conditions such as aging and rheumatoid arthritis [137]. In a patient with relapsed MF, malignant T cells with the same TCR as at initial diagnosis gained CD26/DPP4 expression indicative of evolution of the original malignant clone and highlighting that the loss of CD26/DPP4 expression is not a universal parameter for defining aberrant leukemic T cells in these diseases [138]. Thus, assessment of more clinicopathological features and the refinement of expression thresholds is required for the accurate identification and monitoring of aberrant T cells. Currently, a consortium of EuroFlow is working on the definition of a refined standardized flow cytometry panel assessing the expression of more than eight markers to diagnose SS patients with the highest possible sensitivity.

Of note, also the prognostic value of CD26/DPP4 expression is still a matter of debate. A recent study performed with large-cell transformed MF (a potentially more aggressive variant of MF) patients indicated that reduced CD26/DPP4 expression on CD4^+^ T cells might be associated with poor prognosis [139], whereas a retrospective cohort analysis of 11 patients with erythrodermic CTCL indicated that the frequency of CD26^−^/CD4^+^ T cells was not a reliable marker of treatment response or disease progression [140]. Here, analyses of larger cohorts are needed to evaluate the prognostic value of CD26/DPP4 expression.

In addition, as already mentioned, CD26/DPP4 is stored in the cytotoxic granules in a fraction of cytotoxic T cells and NK cells [22] and its surface expression thus follows a more complex and activation-dependent regulation. However, most if not all studies evaluating the diagnostic or prognostic value of CD26/DPP4 expression were limited to analyses of CD26/DPP4 surface expression.

## 6. Conclusions

CD26/DPP4 is a multifunctional protein with a wide array of substrates and interaction partners. CD26/DPP4 effects are exerted by either the transmembrane protein and/or the soluble form and are dependent or independent of its enzymatic activity. CD26/DPP4-mediated cleavage might result in the modulation of receptor specificities, the inactivation of substrates, or the generation of bioactive peptides. Full length CD26/DPP4 can be released in association with extracellular vesicles, while soluble CD26/DPP4 is shed from the cell surface by proteases. Moreover, CD26/DPP4 is stored in cytotoxic granules of lymphocytes from where it is released upon activation or target cell encounter. Thus, deciphering the precise role of CD26/DPP4 in different aspects of T-cell biology is a challenging task especially with respect to the complex regulation of cytokine/chemokine networks and the likely context-dependent and differential impact of different CD26/DPP4-associated signaling pathways.

Nevertheless, CD26/DPP4 has been clearly implicated in the modulation of T-cell activation, proliferation, and effector function and CD26^+^ T cells are characterized by remarkable anti-tumor properties with respect to cytotoxic and migratory properties. In cutaneous T-cell lymphoma, especially in Sézary syndrome and in mycosis fungoides, CD26/DPP4 expression patterns emerged as an established marker for diagnosis and treatment monitoring.

However, further studies are needed to understand the precise functional role of CD26/DPP4 in the anti-tumor response of T cells to further optimize T-cell based immunotherapies employing CAR T cells or adoptively transferred autologous T cells and to establish the infiltration of different tumor entities with CD26^+^ tumor-infiltrating lymphocytes as a prognostic factor. Likewise, further analyses employing larger patient cohorts might help to further refine the diagnostic value of CD26/DPP4 expression in cutaneous T-cell lymphoma and to assess its suitability as a prognostic factor.

Moreover, whereas some studies already suggest CD26/DPP4 as a therapeutic target in solid cancers, data regarding hematologic malignancies are still missing.

## Data Availability

The data presented in this article are available on request from Marcus Lettau at marcus.lettau@uksh.de.
